# Invariance of the Sexual Double Standard Scale: A Cross-Cultural Study

**DOI:** 10.3390/ijerph17051569

**Published:** 2020-02-29

**Authors:** Maria del Mar Sánchez-Fuentes, Nieves Moyano, Carmen Gómez-Berrocal, Juan Carlos Sierra

**Affiliations:** 1 Department of Social Sciences, Faculty of Human and Social Sciences, University of La Costa, Barranquilla 080002, Colombia; marsanchez@unizar.es; 2 Faculty of Human and Social Sciences, University of Zaragoza, 44003 Teruel, Spain; 3 Faculty of Human Sciences and Education, University of Zaragoza, 22003 Huesca, Spain; 4 Faculty of Humanities and Education Sciences, University of Jaén, 23071 Jaén, Spain; 5 Mind, Brain and Behavior Research Centre, University of Granada, 18011 Granada, Spain; cgomezb@ugr.es (C.G.-B.); jcsierra@ugr.es (J.C.S.)

**Keywords:** Sexual Double Standard, invariance, culture, sex

## Abstract

The Sexual Double Standard (SDS) is an instrument used to judge sexual behavior, in which men are usually granted greater sexual freedom, while the same sexual behavior is condemned in women. Culture can be a relevant variable for the SDS. Therefore, we have examined the measurement invariance of the Sexual Double Standard Scale (SDSS) across the Spanish and Colombian populations, comparing this phenomenon by country and gender. The scale comprises two factors: sexual freedom and sexual shyness. The sample consisted of 1832 heterosexual adults (46.3% men, 53.7% women), 54.3% of whom were Spanish and 45.7% Colombian. Strong invariance was found. The reliability values were good for country and gender. Men and women from both countries supported greater freedom for themselves compared to the other gender. Furthermore, Spanish women, unlike their Colombian counterparts, supported greater sexual shyness for men. Thus, what some authors have labeled as a "reverse sexual double standard" seems to emerge.

## 1. Introduction

Since Reiss [1] conducted his studies on the Sexual Double Standard (SDS), attempts have been made to determine whether similar sexual behaviors are judged differently if they are conducted by men or women. The Sexual Double Standard (SDS) refers to an attitude that implies a different assessment of the same sexual behavior depending on the sex (man vs. woman) of the person exhibiting said behavior. Consequently, SDS contributes to different norms of sexual permissiveness for men and women [2]. In the field of behaviors related to sexual freedom (e.g., sex before marriage, activity with multiple sexual partners, sexual debut at early ages, casual relationships without commitment, or playing an active role in sex), we refer to traditional SDS and the fact that men are allowed more sexual freedom than women [3]. As a result of this traditional SDS, an asymmetric, double standard norm is established, which promotes the approval of sexual behaviors carried out by men and, at the same time, promotes the rejection of those same behaviors when performed by women [4].

### 1.1. Psychological and Social Effects of the Sexual Double Standard

This attitude towards sexual behaviors in heterosexual relationships has both psychological and social implications. On a psychological level, the relevance of its clinical effects is worth highlighting. SDS has been associated with psychological trauma [5], worse sexual health, and an increased risk of contracting sexually transmitted infections [4,6]. From a social point of view, the effect of SDS can take place through macrosocial processes, for example, the social norms that regulate the behavior of men and women, and define what are the appropriate behaviors for each gender, in the field of heterosexual behaviors [7]. The normative role that traditional SDS plays can be verified through studies that relate SDS to sexual victimization [8], and the acceptance of the rape myth, that is, with the denial or minimization of harm to a sexual victim [9].

### 1.2. Culture, Gender, and Sexual Double Standard

From a sociocultural perspective that conceives sexuality as more than the result of biological processes [10], we expect there to be a relationship between personal support for SDS and the culture in which the individual has socialized. In a similar line of reasoning, we also expect support for SDS to be related to the demographic origin of the person. Despite the interest in research provoked by the study of SDS, the samples are usually very homogeneous, both in terms of their sociodemographic characteristics and cultural origin. The authors of a recent meta-analysis on double sexual standard [11] point out the need to work with more diverse samples in terms of age and educational level, culture and ethnicity. In this sense, to improve or expand knowledge about the adherence and prevalence of SDS, it is convenient to collect more diverse samples based on their sociodemographic characteristics, such as gender, age, level of studies, and cultural origin.

The reviews and meta-analyses carried out in recent decades [12,13] show that changes in cultural beliefs about sexual behavior allow us to understand part of the variability observed in the prevalence of SDS. When comparing Spanish-speaking countries (e.g., Spain and Latin American countries), some authors pointed out the prevalence of their own culture in different regions or Spanish-speaking countries, in terms of gender relations [14]. In a broader sense than that of sexist ideology, a comparative study, among Spanish-speaking countries, of gender attitudes towards sexual behaviors should consider the level of individualism-collectivism that characterizes each society. Hofstede [15] coined the term individualism-collectivism (I-C) to refer to the concept of self that a person builds from his/her own culture. In individualistic cultures, the self-concept of each person implies a feeling and awareness of independence regarding the group or groups to which they belong, or with which the person identifies (i.e., in-group). On the contrary, people from collectivist cultures place more importance on the "we" feeling, and the concept of itself is more linked, compared to individualistic societies, to the identification of the person with their membership or reference groups (i.e., in-group). In other words, people from collectivist cultures use their group allegiance as a relevant criterion to define themselves and to value who they are. Since relations between men and women are regulated by gender identity, that is, the identification of men and women with their respective in-groups, it seems logical to assume that—more in individualistic cultures than in collectivist cultures—there is a greater prevalence of different standards or norms regarding what the behavior of men and women should be like in heterosexual encounters. In parallel, there may be less individual adherence to traditional SDS. If we consider the case of Spain and Colombia, Hofstede [15] already stated that Colombia is a country with a low level of individualism. More recent evidence, provided by a study in 56 countries, shows that Spain is a more individualistic country than Colombia [16]. In this study, each country was distributed into a continuous dimension of individualism and collectivism, according to the scores they had obtained in that dimension. The following three aspects were considered: conflict avoidance and conformism, desire for social ascendancy and in-group favoritism vs. out-group exclusionism. The results indicated that Spain ranked 17 (score: 58) and Colombia ranked 44 (score: −78), that is, greater individualism for Spain and greater collectivism for Colombia [14]. The Netherlands ranked in top position, as the most individualistic country (score: 182) and Nigeria, in bottom position (score: −291) as the most collectivist one. Previous studies indicated that Colombian men and women are more erotophobic (i.e., more negative attitudes towards sexuality) than Spanish men and women [17], and that the I-C cultural difference is related to gender differences [18].

With regard to the relationship between sociodemographic characteristics and SDS, it is important to highlight the role that gender plays. It is consistently found that men support attitudes toward traditional SDS, while women tend to support more egalitarian attitudes [8,15]. However, this pattern may be related to the I-C culture to which the person belongs. Recently, Alvarez-Muelas et al. [19] indicated that, in Spain, men reported more traditional SDS than women, for both dimensions of this construct: sexual freedom and sexual shyness. These findings are in line with those reported in similar cultures such as Colombia, as well as El Salvador [20].

As culture can be a relevant variable for SDS, it is necessary to have measures that permit making intercultural comparisons in the adherence and prevalence of SDS. Although a scale or instrument is available in the language spoken by different countries (e.g., Colombia and Spain), this does not ensure the equivalence between the countries of the construct that is measuring the instrument [19,21]. To ensure the equivalence of the SDS construct between groups, whether groups are formed according to the cultural origin of their members, gender or any other demographic criteria, it is necessary to verify the measurement invariance (also known as measurement equivalence) of the SDS. This involves examining whether the construct is evaluated equally in different groups, for example, according to the gender or cultural origin of the sample. However, previously conducted research, comparing different groups by gender, ethnicity, or culture, has not provided guarantees for this measurement invariance, which is a prior step to assessing whether the items measure this attribute equally, without bias [22,23], because if the measurement invariance between the groups is not fulfilled, the interpretations can lead to erroneous conclusions.

One of the most commonly used scales to evaluate SDS is the Sexual Double Standard Scale (SDSS) [24]. The SDSS has been validated in the Spanish population and has adequate psychometric properties [25]. It is also invariant by gender, age and education [19]. However, the psychometric properties of this instrument remain unknown in other countries, for example Colombia, where Spanish is spoken but whose culture is different from what currently exists in Spain.

### 1.3. Current Research

Bearing in mind that measurement invariance (equivalence) is a logical prerequisite for conducting cross-group comparisons, the main objective of the present study was to examine whether the Spanish version of the SDSS of Sierra et al. [25], is cross-culturally invariant between Spain and Colombia. The specific goals of the present study were to analyze the measurement invariance of the SDSS between the Spanish and Colombian populations. We also compared the scores in men and women from both countries to determine the ethnic and gender differences in attitudes toward the roles of men and women in the sexual behavior field.

Accordingly, therefore, we hypothesized the following:

**Hypothesis** **1** **(H1).** *The three-factor structure of the SDSS would show adequate goodness-of-fit indices across samples—from Spain and Colombia—and the SDSS scores would show measurement invariance across samples.*

**Hypothesis** **2** **(H2).** *Spanish individuals would report less support for the traditional SDS than Colombians.*

**Hypothesis** **3** **(H3).** *Men would report more support for the traditional SDS than women.*

## 2. Materials and Methods 

### 2.1. Study Sample and Design

Data were collected from 2118 participants. Of these, 157 were eliminated because they did not answer 25% or more items, and 129 were dropped because they did not meet the inclusion criteria. Thus, the final sample consisted of 1832 heterosexual adults (46.3% men, 53.7% women), 994 (54.3%) of whom were Spanish and 838 (45.7%) were Colombian. Quota sampling was used by considering gender and age subgroups (18–34 years old, 35–49, 50 years old or more). The sample met the following inclusion criteria: (a) being 18 years of age or older; (b) self-identify as heterosexual; (c) have Spanish or Colombian nationality. Socio-demographic information is displayed in Table 1.

### 2.2. Materials

Socio-demographic questionnaire. Information was collected about gender, age, education, nationality, religion, frequency of attendance at religious events, and frequency of prayer in private in places other than their church or place of worship. In addition, participants were asked questions about their sexual orientation, whether they were involved in a relationship at the time of the survey, duration of this relationship, their partner´s age, whether they had sexual activity with their partner, age of the first sexual relationship (oral, vaginal, and/or anal), and number of sexual partners in their lifetime.

Sexual Double Standard Scale (SDSS). The Spanish version of Sierra et al. [25] comprised 16 items distributed into two dimensions (Acceptance for sexual freedom and Acceptance for sexual shyness), with a 4-point Likert scale ranging from 0 (strongly disagree) to 3 (strongly agree). The scale presented adequate internal consistency reliability; 0.84 for Acceptance for sexual freedom and 0.87 for Acceptance for sexual shyness. Its test-retest reliability—the scale was administered to the same respondents three times—ranged from 0.71 and 0.78 (after 8 weeks and 4 weeks, respectively), establishing adequate evidence for validity with other measures such as social dominance orientation [26]. The sum of the scores from the eight items in each subscale provides two SDS indices, one Index of Double Standard for Sexual Freedom (IDS-SF) and another Index of Double Standard for Sexual Shyness (IDS-SS). For the IDS-SF, some items related to women are reversed, while some items related to men are reversed for the IDS-SS. Higher index scores indicate a higher traditional SDS. For each index, scores can range from −12 to +12. For the IDS-SF, scores close to −12 indicate being in favor of more sexual freedom for women than for men, and scores close to + 12 indicate being in favor of more sexual freedom for men than for women. For the IDS-SS, scores close to −12 indicate being in favor of more sexual shyness in men than in women and scores close to +12 indicate being in favor of more sexual shyness in women than in men.

### 2.3. Procedure

This study was approved by the Ethics Committee on Human Research of the University of Granada, Spain. Non probabilistic quota sampling was used with the general population in Spain and Colombia. Participants were invited to participate in an online survey. The URL was distributed on different social networks and via the news service of the University of Granada between February and July 2018. To access the survey, participants had to confirm access by answering a security question consisting of a random sum. In the responses, the IP of the device was controlled to prevent the same participant from answering more than once. At the beginning of the survey, informed consent forms were distributed, which included the main study objective and the approximate time to complete the measures. The participants took approximately 20 minutes to complete the survey. Anonymity and confidentiality were guaranteed. All participants were volunteers and did not receive any compensation for taking part in this study.

## 3. Data Analysis

Measurement invariance was examined in order to test whether the factor structure of the measure was equivalent across both countries, i.e., Spain and Colombia. The factor structure previously validated by Sierra et al. [25] was tested; that is: Factor 1: Acceptance for sexual freedom (ASF; items 1, 2, 3, 4, 6, 11, 13, and 14); Factor 2: Acceptance for sexual shyness (ASS; items 5, 7, 8, 9, 10, 12, 15, and 16). Byrne’s recommendations [27] were followed for the analysis. Version 24 of the AMOS software was used. The Maximum Likelihood (ML) method was followed. Indication of invariance was considered if the increased Comparative Fit Index (CFI) value was higher than 0.01, as well as an increase of χ^2^ /*gl* in relation to the previous model [28]. Reliability values were obtained with McDonald´s index ω for each dimension according to country and gender. Finally, the existence of significant differences in the SDS scores according to country, gender, and the interaction of both was tested, through MANOVA, by comparing the scores obtained in the IDS-SF and the IDS-SS.

## 4. Results

### 4.1. Measurement Invariance

**Figure 1:**
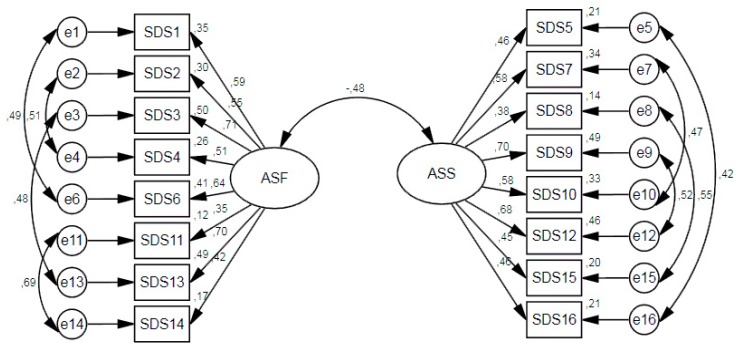
Standardized loadings of the factor structure in the Spanish sample.

**Figure 2:**
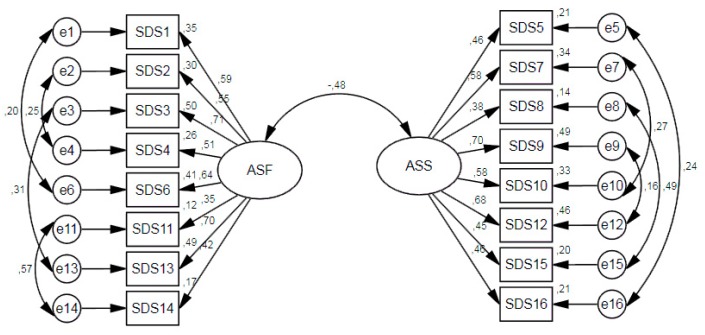
Standardized loadings of the factor structure in the Colombian sample.

In a preliminary analysis, the goodness-of-fit indices were not optimal, so the modification indices were observed. They indicated the need to correlate the following pairs of items: 1–6, 2–4, 3–13, and 11–14 for ASF, and 5–16, 7–10, 8–15, and 9–12 for ASS. These modifications were performed considering similarities in the content of the items, as the pair of items to be correlated included the same content, and referred to either men or women (e.g., item 1: It’s okay for a woman to have more than one sexual relationship at the same time) (e.g., item 6: It’s okay for a man to have more than one sexual relationship at the same time). As shown in Table 2, strong invariance was found. The standardized coefficients of the factor structure for each dimension are shown in Figure 1 and Figure 2 for the Spanish and the Colombian samples, respectively.

### 4.2. Reliability

The reliability of each dimension was obtained separately for men and women, for each country, through McDonald’s ω. For the ASF dimension, the values in the Spanish sample were 0.80 for men and 0.83 for women. In the Colombian sample, the values were 0.82 for men and 0.83 for women. Regarding the ASS dimension, in the Spanish sample a value equal to 0.81 was obtained for men and 0.86 for women. In the Colombian sample, the values were 0.73 for men and 0.78 for women.

### 4.3. Differences Across Country and Gender in the SDSS Indices

MANOVA was performed to examine whether there were significant differences in the indices of the SDSS for country and gender. In the samples of both countries, men, opposed to women, obtained higher positive IDS-SF scores, which meant that they, more than women, endorsed greater sexual freedom for themselves than for women. However, women from both countries obtained negative scores, which indicated a tendency to support greater sexual freedom for women (vs. men). Regarding the sexual shyness index, in the samples from both countries, men (vs. women) obtained positive and higher scores; that is, men (vs. women) expressed they were in favor of more sexual shyness for women than for men. Interestingly, Spanish women obtained negative sexual shyness scores, while Colombian women scored positively. This indicates that Spanish women support sexual restraint for men to a greater extent than for women, while Colombian women support greater sexual restraint for women; that is, they manifest a similar attitude to that maintained by men for this issue (see Table 3).

Considering the scores obtained by country, in the Spanish sample, scores close to 0 were obtained in both indices; that is, for both sexual freedom and sexual shyness, which did not indicate a tendency to support double standards. However, positive values were obtained in both indices in the Colombian sample. This indicated a tendency to support more sexual freedom for men than for women—although effect size was low—and more sexual restraint for women than for men; that is, in both indices, support for the traditional double sexual standard was found. Finally, a significant interaction emerged between country and gender in the sexual freedom index. Here, three aspects should be stressed: (a) men from both countries supported greater sexual freedom for their endogroup than for the group of women; (b) women from both countries supported greater sexual freedom for their own group that for the group of men; (c) the differences in support for greater sexual freedom that men and women from each country granted their own endogroup were bigger for Colombia than for Spain; that is, between both genders, the defense of endogroup favoritism in sexual freedom was more polarized in Colombia than in Spain.

## 5. Discussion

This research analyzed the equivalence of the factor structure of the SDSS, which permits evaluating the double sexual standard between two culturally different countries: one more individualistic (Spain) and another more collectivist (Colombia). The SDSS scale provides a measure of SDS in two areas of sexual behavior: ​​sexual freedom and sexual shyness. In order to closely examine the role of culture in attitudes toward SDS, the differences between both countries were examined in-depth, in addition to gender comparisons. Our findings allowed to support the structural equivalence of the scale between Spain and Colombia with the covariance of errors in the scores of the paired items. Moreover, a more favorable attitude to the traditional double sexual standard was found in Colombia than in Spain.

### 5.1. An Invariant Measure of the Sexual Double Standard

First, the structural equivalence of the scale was obtained, which provides guarantees to make comparisons across the scores of participants from both countries with a low measurement bias [21,29]. That is, the SDSS is invariant in culturally different countries like Spain and Colombia. Therefore, this research overcomes a major limitation compared to previous research in which the equivalence of the scales across countries was uncertain. In addition, the present study ratified the appropriate psychometric properties of the SDSS, which can be used in different cultures, so that knowledge of the double sexual standard could be improved [25].

### 5.2. More Sexual Freedom for the In-Group

Our findings showed that, in both countries, men supported the traditional double standard; that is, they still maintained their attitude in favor of more sexual freedom for men than for women, and more reluctance for women than for men. However, these patterns in women seemed to have changed. The Spanish and Colombian women reported endorsing more sexual freedom for women than for men. These results are congruent with the progressive reduction of attitudes that support a traditional double sexual standard [30]. In line with this, a fourth wave is arising worldwide in defense of women and feminism. This wave is characterized by a wide range of women’s attitudes and behaviors to defend their rights, feelings of greater freedom, autonomy and the visibility of feelings of support, so as not to be afraid of being a woman, through #Metoo movements against violence and sexual assault.

### 5.3. Cultural Gap between Women

However, differences between the Spanish and Colombian women were found in terms of adherence to SDS in the area of ​​sexual shyness. Spanish women supported sexual restraint to a greater extent for men than for women (e.g., reverse SDS), while Colombian women supported greater sexual shyness for women than for men (e.g., traditional SDS). However, men seemed to endorse patriarchal roles. In both countries, the results relating to men are similar to previous studies. In addition, as in other studies, the responses of women in the field of sexual freedom indicate attitudes favorable to reverse SDS or sexual equality, in both countries [31]. Unlike other studies that worked with Western samples (i.e., individualists), we have found a pattern of results that may be indicative of the role of culture. Specifically, we have found that Colombian women, in the field of sexual shyness, support a traditional SDS (e.g., defense of more sexual shyness for women than for men). This result could be explained because in Colombia, as in other Latin American countries, there is more sexism, both hostile and benevolent [32]. Since sexism is a prejudiced attitude that acquires meaning in the context of intergroup relations between men and women, it contributes to asymmetrically defining the gender identity of women and men in terms of privileges, in different areas of social behaviors, and also in the area of sexuality. In agreement with this approach, in more collectivist countries, such as Colombia, women take female gender identity more into consideration, largely based on social consensus, to define and internalize what sexual behaviors they can or cannot perform. Future research must analyze, on the one hand, whether benevolent sexism may be favoring this traditional SDS in the field of sexual shyness. On the other hand, the role of the norms on heterosexual roles in the support and prevalence of traditional SDS—egalitarian or reverse—both in the area of freedom and of sexual shyness should be studied. Therefore, future research is recommended to examine the perception of support for the double sexual standard in Colombian society by, for example, adapting and validating the SDSS as a macropsychological indicator, which has already been done in Spain [7]. From a macropsychological level of analysis, the respondents of the scale receive the following instruction when responding to the items: “We would like to know to what extent you believe that most people agree or disagree with each of the following statements”. Therefore, the responses to this question would better reflect the subject´s perceptions of the sexual societal double standard as it examines the individual´s perceptions of what others think about this topic. Both self-referred and hetero-referred (macropsychological indicator) versions of the scale provide information about an individual´s endorsement of the double standard, an individual´s perception of how society endorses the double standard, and an individual´s conformity to the social norms that (s)he perceives.

### 5.4. Sexual Double Standard: Cultural and Gender Differences

Regarding differences by country, Colombian participants of both genders supported greater sexual freedom for men and greater shyness for women, with statistically significant differences appearing when these results were compared with Spanish participants. According to our hypothesis, we conclude that there is more support for the traditional double standard in Colombia. A previous study concluded that Colombian individuals showed greater erotophobia than Spaniards [17]. 

In addition, a significant interaction between sex and country was found regarding sexual freedom. Both men and women from both countries supported greater sexual freedom for their endogroup, thus indicating endogroup favoritism. This result is not surprising if we consider that people tend to favor their own sex [33]. In addition, by taking into account the differences between countries, endogroup favoritism was stronger in Colombia than in Spain. Indeed, endogroup favoritism has been related to more sexist attitudes [34] and Colombia is a more sexist country than Spain [35], which could explain this difference across countries. 

### 5.5. Emergence of a Reverse Form of the Sexual Double Standard

In summary, our findings for men indicated that they still supported the traditional double standard, and this pattern is consistent with previous studies [4,36]. However, for the sample from Spain, but not from Colombia, our findings indicated support for the recently so-called “reverse” sexual double standard; that is, more sexual reluctance for men than for women. Previous studies have shown a more negative assessment toward men (vs. women) when they expressed assertive sexual behavior [37]. In contrast, the manifestation of sexual initiative has been more favorably evaluated in women than in men [38]. These results may, at least in part, be determined by current media pressure and due to the emerging awareness against men’s gender violence toward women. Certain sexual behaviors that were “praised” or “rewarded” in men have now acquired a negative nuance, and have been labeled as a possible risk of “sexual predator” [2]. Future studies should analyze the role of these two factors in the change of attitudes toward SDS that women are beginning to express: the fight against gender violence and the fight for democracy in gender relationships. While both factors would facilitate the promulgation of egalitarian norms in the sexual behavior field, differences and conflicts between men and women could be reduced if the defense of sexual equality is motivated by the desire to achieve a more equitable and democratic society in all areas.

### 5.6. Limitations and Future Research

The present study has some limitations. First, as non-probabilistic sampling was used, the results obtained cannot be generalized to the general population. Second, no strict invariance of the scale was reached, thus the measure is not free of bias. Third, external validity was not examined, so the extent to which the self-reported attitudes in the SDSS really reflect an individual´s behavior remains unknown. Therefore, future studies using representative samples and analyzing evidence for external validity of the scale are recommended. Future research should also analyze the double sexual standard in sexual minorities as very little research has been conducted in these populations [39].

## 6. Conclusions

We conclude that the SDSS has adequate psychometric properties and is an equivalent scale to be used in Colombia and Spain. It is worth mentioning that maintaining sexual patterns based on the double sexual standard has several negative consequences, such as increased women´s objectification and a higher risk of aggression and violence [8]. On the contrary, supporting egalitarian or feminist attitudes has a number of positive outcomes, such as reduction of sexism or rejection of objectification [40]. Future studies should assess the degree to which subjects self-identify with egalitarian ideas or values, as the identification leads to willingness to develop egalitarian behaviors, as derived from studies conducted on feminist identification. In addition, both the endorsement and expression of sexism in both public and private spheres should be more deeply explored, as some differences have been found [41], as well as how self-reported measures really capture both dimensions.

## Figures and Tables

**Table 1:** The sample’s socio-demographic characteristics.

	Spain					Colombia				
Variables	Men		Women			Men		Women		
	*n*	%	*n*	%	*x^2^*	*n*	%	*n*	%	*x^2^*
Age					0.29					2.04
18–34	263	54.6	287	56.1		236	64.0	321	67.4	
35–49	165	34.2	166	32.4		60	16.3	76	16.0	
≥50	54	11.2	59	11.5		73	19.8	79	16.6	
Education					25.03 ***					7.86
Uneducated	-	-	-	-		1	0.3	3	0.6	
Primary	3	0.6	31	6.1		6	1.6	16	3.4	
Secondary	99	20.6	79	15.4		85	23.1	81	17.0	
University	378	78.8	402	78.5		276	75.0	376	79.0	
Attendance at religious events					2.63 **					2.19 *
Never	181	37.8	93	18.5		58	15.7	47	9.9	
Less than once a month	177	37.0	285	56.5		67	18.2	84	17.6	
Once a month	30	6.3	18	3.6		33	8.9	42	8.8	
A few times a month	48	10.0	56	11.1		65	17.6	89	18.7	
Once a week	25	5.2	32	6.3		67	18.2	97	20.4	
A few times a week	11	2.3	16	3.2		60	16.3	92	19.3	
Once a day	3	0.6	1	0.2		14	3.8	14	2.9	
More than once a day	4	0.8	3	0.6		5	1.4	11	2.3	
Praise frequency					4.68 ***					5.81 ***
Never	255	52.9	177	35.0		45	12.2	27	5.7	
Less than once a month	76	15.8	107	21.2		30	8.2	20	4.2	
Once a month	12	2.5	11	2.2		10	2.7	12	2.5	
A few times a month	37	7.7	58	11.5		45	12.2	33	6.9	
Once a week	10	2.1	11	2.2		19	5.2	13	2.7	
A few times a week	39	8.1	56	11.1		56	15.2	75	15.8	
Once a day	34	7.1	57	11.3		96	26.1	171	36.0	
More than once a day	16	3.3	28	5.5		67	18.2	124	26.1	
Currently in a relationship										
Yes	390	83.0	368	73.3		293	79.8	401	84.2	
No	80	17.0	134	26.7		74	20.2	75	15.8	
Mean age at first sexual intercourse *(SD)*	434	17.69 (3.30)	431	18.01 (3.42)	−1.41ns	358	16.30 (2.84)	441	18.29 (3.05)	−9.47 ***
Mean number of sexual partners *(SD)*	433	5.58 (6.70)	438	3.98 (4.66)	4.09 ***	358	6.91 (7.90)	443	3.43 (4.72)	7.71 ***

*Note*. *** *p* < 0.001; ** p < 0.01; * *p* < 0.05.

**Table 2:** Goodness-of-fit indices for measuring invariance across Spain and Colombia.

	χ^2^/*gl*	RMSEA	TLI	CFI
Configural invariance	6.20	0.053	0.89	0.91
Weak invariance	6.14	0.053	0.89	0.90
Strong invariance	6.17	0.053	0.89	0.90
Strict invariance	6.68	0.056	0.88	0.88

*Note.* RMSEA: Root Mean Square Error of Approxiamation; TLI: Tucker-Lewis Index; CFI: Comparative Fit Index.

**Table 3:** Descriptive statistics and differences by MANOVA in the Sexual Double Standard (SDS) indices for country, gender, and their interaction.

	Spain					Colombia							
	Men	Women	Global Sample			Men	Women	Global Sample			Country		Country x Sex
	*M (SD)*	*M (SD)*	*M (SD)*	*F*_(1,992)_	*d*	*M (SD)*	*M (SD)*	*M (SD)*	*F*_(1,836)_	*d*	*F*_(1,1830)_	*d*	*F*
IDS-SF	0.41 (1.72)	−0.37 (1.40)	0.00 (1.61)	62.66 ***	0.49	0.90 (2.14)	−0.09 (1.54)	0.34 (1.90)	61.69 ***	0.53	16.62 ***	0.19	10.12 ***
IDS-SS	0.40 (1.75)	−0.25 (1.37)	0.06 (1.60)	47.84 ***	0.41	1.08 (2.30)	0.44 (1.80)	0.72 (2.06)	20.41 ***	0.30	60.23 ***	0.35	0.98

*Note*. *d* refers to Cohen´s *d*; *** *p* < 0.001.

## References

[1] Reiss I.L. (1964). Premarital Sexual Standards in America a Sociological Investigation of the Relative Social and Cultural Integration of American Sexual Standards.

[2] Milhausen R.R., Herold E.S. (2002). Reconceptualizing the sexual double standard. J. Psychol. Hum. Sex.

[3] Fasula A.M., Carry M., Miller K.S. (2014). A multidimensional framework for the meanings of the sexual double standard and its application for the sexual health of young black women in the US. J. Sex Res..

[4] Marks M.J., Young T.M., Zaikman Y. (2018). The sexual double standard in the real world: Evaluations of sexually active friends and acquaintances. Soc. Psychol..

[5] Farvid P., Braun V., Rowney C. (2017). ‘No girl wants to be called a slut!’: Women, heterosexual casual sex and the sexual double standard. J. Gend. Stud..

[6] Ramiro-Sánchez T., Ramiro M.T., Bermúdez M.P., Buela-Casal G. (2018). Sexism and sexual risk behavior in adolescents: Gender differences. Int. J. Clin. Health Psychol..

[7] Gómez Berrocal C., Vallejo-Medina P., Moyano N., Sierra J.C. (2019). Sexual Double Standard: A psychometric study from a macropsychological perspective among the Spanish heterosexual population. Front. Psychol..

[8] Eaton A.A., Matamala A. (2014). The relationship between heteronormative beliefs and verbal sexual coercion in college students. Arch. Sex Behav..

[9] Klement K.R., Sagarin B.J., Lee E.M. (2017). Participating in a culture of consent may be associated with lower rape-supportive beliefs. J. Sex Res..

[10] Simon W., Gagnon J.H. (2003). Sexual scripts: Origins, influences and changes. Qual. Sociol..

[11] Endendijk J.J., van Baar A.L., Deković M. (2019). He is a Stud, She is a Slut! A Meta-Analysis on the Continued Existence of Sexual Double Standards. Pers. Soc. Psychol. Rev..

[12] Bordini G.S., Sperb T.M. (2013). Sexual double standard: A review of the literature between 2001 and 2010. Sex. Cult..

[13] Wells B.E., Twenge J.M. (2005). Changes in young people’s sexual behavior and attitudes, 1943–1999: A cross-temporal meta-analysis. Rev. Gen. Psychol..

[14] Rodríguez A., Marín L., Leone M.E. (1993). The machismo in the social imaginary. Lat. Am. J. Psychol..

[15] Hofstede G. (2001). Cultures Consequences. Comparing Values, Behaviors, Institutions and Organizations across Cultures.

[16] Minkov M., Dutt P., Schachner M., Morales O., Sanchez C., Jandosova J., Mudd B. (2017). A revision of Hofstede’s individualism-collectivism dimension: A new national index from a 56-country study. Cross Cult. Strateg. Manag..

[17] Vallejo-Medina P., Marchal-Bertrand L., Gómez-Lugo M., Espada J.P., Sierra J.C., Soler F., Morales A. (2016). Adaptation and validation of the Brief Sexual Opinion Survey (SOS) in a Colombian sample and factorial equivalence with the Spanish version. PLoS ONE.

[18] Ubillos S., Zubieta E., Páez D., Deschamps J.C., Ezeiza A., Vera A. (2001). Amor, cultura y sexo [Love, culture and sex]. Rev. Electron. Motiv. Emoc..

[19] Álvarez-Muelas A., Gómez Berrocal C., Vallejo-Medina P., Sierra J.C. (2019). Invariance of Spanish version of Sexual Double Standard Scale across sex, age, and education level. Psicothema.

[20] Gutiérrez-Quintanilla J.R., Rojas-García A., Sierra J.C. (2010). Comparación transcultural de la doble moral sexual entre estudiantes universitarios salvadoreños y españoles [Transcultural comparison of double sexual standard among Salvadorian and Spanish undergraduate students]. Rev. Salv. Psicol..

[21] Muñiz J., Elosua P., Hambleton R.K. (2013). Directrices para la traducción y adaptación de los tests: Segunda edición [Guidelines for the translation and adaptation of tests]. Psicothema.

[22] Mellenbergh G.J. (1989). Item bias and item response theory. Educ. Res..

[23] Meredith W. (1993). Measurement invariance, Factor-Analysis and Factorial Invariance. Psychometrika.

[24] Muehlenhard C.L., Quackenbush D.M., Fisher T.D., Davis C.M., Yarber W.L., Davis S.L. (1998). Sexual Double Standard Scale. Handbook of Sexuality Related Measures.

[25] Sierra J.C., Moyano N., Vallejo-Medina P., Gómez Berrocal C. (2018). An abridged Spanish version of Sexual Double Standard Scale: Factorial structure, reliability and validity evidence. Int. J. Clin. Health Psychol..

[26] Pratto F., Sidanius J., Stallworth L.M., Malle B.F. (1994). Social dominance orientation: A personality variable predicting social and political attitudes. J. Pers. Soc. Psychol..

[27] Byrne B.M. (2003). Structural Equation Modeling with Lisrel, Prelis, and Simplis: Basic Concepts, Applications, and Programming.

[28] Cheung G.W., Rensvold R.B. (2002). Evaluating goodness-of-fit indexes for testing measurement invariance. Struct. Equ. Model..

[29] Dimitrov D.M. (2011). Testing for factorial invariance in the context of construct validation. Meas Eval Couns Dev.

[30] Petersen J.L., Hyde J.S. (2011). Gender differences in sexual attitudes and behaviors: A review of meta-analytic results and large datasets. J. Sex Res..

[31] Allison R., Risman B.J. (2013). A double standard for “Hooking Up”: How far have we come toward gender equality?. Soc. Sci. Res..

[32] Vaamonde J.D., Omar A. (2017). Perceptions of organizational justice and ambivalent sexism: The moderation role of individualism-collectivism. Rev. Psicol..

[33] Rudman L.A., Goodwin S.A. (2004). Gender differences in automatic in group bias: Why do women like women more than men like men?. J. Pers. Soc. Psychol..

[34] Gómez-Berrocal C., Cuadrado I., Navas M., Quiles M.N., Morera M.D. (2011). Sexismo hostil y benevolente: Dimensiones de comparación intergrupal, imagen de los subtipos de mujer y autoimagen del endogrupo [Hostil and benevolent sexism: Dimensions of intergrupal comparison, subtypes women’s image and self-image from the endrogroup]. Rev. Psicol. Soc..

[35] Moya M., Páez D., Glickndez P., Fernández I., Poeschl G. (2002). Masculinidad-feminidad y factores culturales [Masculinity-feminity and cultural factors]. Rev. Esp. Motiv. Emoc..

[36] Kreager D.A., Staff J. (2009). The sexual double standard and adolescent peer acceptance. Soc. Psychol. Quart..

[37] Klein V., Imhoff R., Reininger K.M., Briken P. (2019). Perceptions of Sexual Script Deviation in Women and Men. Arch. Sex Behav..

[38] Thompson A.E., Hart J., Stefaniak S., Harvey C. (2018). Exploring heterosexual adults’ endorsement of the sexual double standard among initiators of consensually nonmonogamous relationship behaviors. Sex Roles.

[39] Calvillo C., Sánchez-Fuentes M.M., Sierra J.C. (2018). Revisión sistemática sobre la satisfacción sexual en parejas del mismo sexo [Systematic review of sexual satisfaction in same-sex couples]. Rev. Iberoam. Psicol. Salud.

[40] Borowsky H.M., Eisenberg M.E., Bucchianeri M.M., Piran N., Neumark-Sztainer D. (2016). Feminist identity, body image, and disordered eating. Eat. Disord..

[41] Chisango T., Mayekiso T., Thomae M. (2015). The social nature of benevolent sexism and the antisocial nature of hostile sexism: Is benevolent sexism more likely to manifest in public contexts and hostile sexism in private contexts?. Int. J. Psychol..

